# Supplementary material for: Resolving the full spectrum of human genome variation using Linked-Reads
Source: Genome Res. 2019 Apr;29(4):635–45. doi: 10.1101/gr.234443.118 (PMC6442396; doi:10.1101/gr.234443.118)
Supplement: Supplemental Material [file supp_gr.234443.118_Supplemental_longranger-2.2.zip › longranger-2.2.tar.bz2!/lr2.2-public/lib/stan/cmdstan/stan/doc/api/html/math_2matrix_2trace__gen__inv__quad__form__ldlt_8hpp.html]

Stan: src/stan/math/matrix/trace\_gen\_inv\_quad\_form\_ldlt.hpp File Reference


|  |  |
| --- | --- |
|  | Stan  2.5.0  probability, sampling & optimization |


- Main Page
- Related Pages
- Namespaces
- Classes
- Files

- File List
- File Members

All Classes Namespaces Files Functions Variables Typedefs Enumerations Enumerator Friends Macros Pages

- src
- stan
- math
- matrix

Namespaces |
Functions

trace\_gen\_inv\_quad\_form\_ldlt.hpp File Reference

`#include <stan/math/matrix/Eigen.hpp>`  
`#include <stan/math/matrix/LDLT_factor.hpp>`  
`#include <stan/math/error_handling/matrix/check_multiplicable.hpp>`  
`#include <stan/math/error_handling/matrix/check_square.hpp>`  
`#include <stan/math/matrix/mdivide_left_ldlt.hpp>`  
`#include <stan/math/matrix/trace.hpp>`  
`#include <stan/math/matrix/transpose.hpp>`  
`#include <stan/math/matrix/multiply.hpp>`

Go to the source code of this file.

|  |  |
| --- | --- |
| Namespaces | |
|  | stan |
|  | Probability, optimization and sampling library. |
|  | |
|  | stan::math |
|  | Matrices and templated mathematical functions. |
|  | |

|  |  |
| --- | --- |
| Functions | |
| template<typename T1 , typename T2 , typename T3 , int R1, int C1, int R2, int C2, int R3, int C3> | |
| boost::enable\_if\_c  <!stan::is\_var< T1 >::value   &&!stan::is\_var< T2 >::value   &&!stan::is\_var< T3 >::value,   typename   boost::math::tools::promote\_args  < T1, T2, T3 >::type >::type | stan::math::trace\_gen\_inv\_quad\_form\_ldlt (const Eigen::Matrix< T1, R1, C1 > &D, const stan::math::LDLT\_factor< T2, R2, C2 > &A, const Eigen::Matrix< T3, R3, C3 > &B) |
|  | |


---

[ Stan Home Page ]

*© 2011–2014,
Stan Development Team.*
